# 支气管镜介入治疗肺肉瘤

**DOI:** 10.3779/j.issn.1009-3419.2016.09.07

**Published:** 2016-09-20

**Authors:** 洪武 王, 楠 张, 冬妹 李, 珩 邹, 洁莉 张, 云芝 周, 秀云 白

**Affiliations:** 1 100028 北京，煤炭总医院呼吸内科 Department of Respiratory Medicine, Meitan General Hospital, Beijing 100028, China; 2 100028 北京，煤炭总医院肿瘤内科 Department of Medical Onology, Meitan General Hospital, Beijing 100028, China

**Keywords:** 肺肉瘤, CT, 介入治疗, Pulmonary sarcoma, Computed tomography, Interventional therapy

## Abstract

**背景与目的:**

肺肉瘤为发生于肺部少见的软组织恶性肿瘤，常规治疗首选手术。本研究旨在探讨采用支气管镜介入治疗的效果。

**方法:**

回顾性分析2008年11月-2014年7月我院收治的16例肺肉瘤患者，平均年龄（53.1 ±5.4）岁。所有患者首次治疗均选用全凭静脉麻醉下硬质气管镜检查，发现肿瘤后行支气管镜介入治疗。再次检查时行电子支气管镜检查。

**结果:**

16例患者中肉瘤样癌10例，纤维肉瘤和肉瘤各2例，纤维粘液性肉瘤和梭性细胞型滑膜肉瘤各1例。周围型11例（占68.8%），主要位于右上叶和左下叶肺内；中央型5例（31.2%）。位于肺内者以混合型多见（9/11, 82%），原发性较多（9/11, 82%）；而位于大气道者以管内型多见（9/16, 56%），转移性较多（11/16, 69%）。支气管镜介入治疗后4例全肺不张均缓解，7例肺叶不张3例完全缓解，部分缓解和无效各2例。患者气道阻塞、卡氏体力状态（Karnofsky performance status, KPS）和气促评分均有明显改善。

**结论:**

支气管镜介入治疗能快速、有效地清除气道内肿瘤，缓解气道梗阻，改善症状。

## Abstract (en)

**Background and objective:**

Pulmonary sarcoma is a rare malignant tumor in soft tissues. Resection is the preferred option to treat this tumor. The aim of this study is to explore the effect of interventional bronchoscopies in the treatment of pulmonary sarcoma if the patient is inoperable.

**Methods:**

Sixteen cases with pulmonary sarcoma were retrospectively reviewed in our hospital from November 2008 to July 2014. The mean age was (53.1±5.4) years old. Rigid bronchoscopy was applied for the first procedure with general anesthesia, and electronic bronchoscopy was used for the second procedure or slight patients.

**Results:**

Sixteen cases, which include 10 sarcomatoid carcinoma, 2 fibrosarcoma, 2 sarcoma, 1 fibromucoid sarcoma, and 1 spindle cell synovial sarcoma, were collected in this study. Eleven cases (68.8%) were peripheral and mainly located in the right upper lobe and left lower lobe. Five cases (31.2%) were central. Of these cases, 82% (9/11) were mixed and primary tumors in pulmonary tumor. Meanwhile, 56% (9/16) were intraluminal and 69% (11/16) were metastatic in central airway. All of the four cases with whole atelectasis were completely relieved through postbronchoscopic interventions. Three of the seven cases with segment atelectasis were completely reopened; two of them were partially relieved; and the remaining two had no response. The obstructive degree, Karnofsky performance status (KPS), and shortness of breathless score improved significantly after the treatment.

**Conclusion:**

Interventional bronchoscopy could rapidly and efficiently remove endobronchial tumor, relieve airway obstruction, and improve clinical symptoms.

原发性肺肉瘤是一种极少见的肺部原发性恶性肿瘤^[1]^，起源于肺间叶组织，极易误诊。根据病理又可分为肺肉瘤样癌、肉瘤（纤维组织肉瘤^[2]^、梭形细胞型肉瘤^[3]^）等。一般原发于肺组织，大气道内罕见^[1-4]^。手术切除是肺肉瘤的主要治疗方法^[1]^，而介入治疗方面的文献报道非常稀缺。作者总结近年来经支气管镜介入治疗的16例肺肉瘤患者，能为国内呼吸介入治疗肺肉瘤提供一定的诊疗意见。供临床分享，以提高认识。

## 1 资料与方法

### 1.1 患者资料

选取2008年11月2014年7月我院收治的肺肉瘤患者16例，平均年龄（53.1±5.4）岁。男性10例[（56.1±2.8）岁]，女性6例[（31.3±8.1）岁]。其中肉瘤样癌10例，纤维肉瘤和肉瘤各2例，纤维粘液性肉瘤和梭形细胞型滑膜肉瘤各1例。所有患者支气管镜检查前后均行常规胸部计算机断层扫描（computed tomography, CT）检查，最后均经病理确诊。根据肿瘤-淋巴结-转移（tumor-node-metastasis, TNM）临床分期，Ⅱb期1例，Ⅲa期7例、Ⅲb期1例、Ⅳ期7例。

参照文献^[5]^，作者将气管内肿瘤胸部CT所见分为四种类型：管内型、管壁型、管外型和混合型。①管内型：为广基底结节或肿块型，肿物呈息肉或结节状突向腔内，呈宽基底贴附于管壁，瘤体与气管壁分界不清，伴管壁局限性增厚，管腔变窄。瘤体与管壁夹角成钝角。管腔、管壁受累范围常大于180°。②管壁型：沿管壁浸润状增厚型，肿瘤起源于气管黏膜上皮及腺体组织，并沿管壁长轴浸润生长，使管壁全层、全周或近全周增厚，致管腔重度狭窄。③管外型：管腔内外生长型，为肿瘤穿破管壁向腔外生长，轮廓不规则或分叶。向腔内生长为主者管腔明显狭窄，若向腔外生长，常累及纵隔及颈部结构。④混合型：可以为前3种形式的任意两种以上病变的组合。

根据作者的经验^[6]^，又将中央型气道分为8个区：主气管等分为3部分，自上而下为1、2、3区；纵隔为4区；右主支气管为5区；右中间段支气管为6区；左主支气管近1/2段为7区，远1/2段为8区。不同的区域应采取治疗的方法不同。根据侵犯范围，将病变分为局限性和弥漫性。局限性为指侵犯1个区的病变，弥漫性指侵犯2个区以上的病变。患者身体状况和气促情况分别采用卡氏体力状态（Karnofsky performance status, KPS）和气促评分法^[7]^。

### 1.2 支气管镜检查

根据病情轻重和胸部CT表现，所有患者首次治疗均选用全凭静脉麻醉下硬质气管镜检查，所用硬质气管镜为德国Tutlingen产品Karl Storz。所用电子气管镜为日本PENTAX公司产品EPM 3500。硬质气管镜术前做胸部CT（必要时行增强CT扫描）及肺功能，由内镜医师及麻醉师进行评估。术中需监测血氧饱和度、心电图、血压及呼吸运动等。

患者平卧手术床上，肩背部底下放一垫子，以使头后仰，便于硬质镜插入。充分氧合好后给予全凭静脉麻醉。经口插入硬质气管镜，通过硬质气管镜的侧孔连接麻醉机或高频喷射通气，维持足够的氧饱和度。同时，在不停呼吸机的情况下通过硬质气管镜后孔进行各种操作。术中通过电子气管镜活检孔插入氩等离子体凝固（argon plasma coagulation, APC）电极、冷冻探头、电圈套器等设备进行介入治疗。

对病情较轻的患者或非首次治疗则单纯在电子气管镜下进行治疗，所有患者均采用监控下监控静脉麻醉，毋须用呼吸机机械通气。操作按常规进行。

### 1.3 气管镜介入治疗

APC所用设备为德国产CESEL 3000型。将APC探针通过电子气管镜活检孔伸出气管镜插入端（能见到探针标志为准），在距病灶0.5 cm以内时开始烧灼。APC输出功率为30 W-50 W，氩气流量为0.8 L/min-1.6 L/min。烧灼过程中勿需停止吸氧，但吸氧浓度需低于40%，持续时间不能太长，并不断用活检钳取出碳化凝固的组织（碳化的组织易燃着火）。

冷冻机采用北京库兰医疗设备有限公司生产的冷冻治疗仪K300型和德国ERBE。被膜金属支架为江苏西格玛公司生产。软式可弯曲冷冻探头直径1.9 mm-2.3 mm，探针末端长度5 mm。冷源为液态二氧化碳。将冰冻探头的金属头部放在肿瘤表面或推进到肿瘤内，冷冻5 s-10 s，使其周围产生最大体积的冰球，在冷冻状态下将探头及其粘附的肿瘤组织取出，必要时再插入探头，直至将腔内的肿瘤全部取出。冻取后如有出血，则结合APC止血。若将冰冻探头的金属头部放在病灶表面持续冷冻1 min-3 min，称为冻融。

电圈套器为南京微创公司生产。将电圈套器连接在高频电刀上。通过电子气管镜的活检通道将电圈套器套扎在肿瘤上，然后启动高频电凝，将肿瘤切下。再用光学活检钳或冷冻将切下的肿瘤取出。

放射性粒子植入：在气管镜引导下，通过WANG氏穿刺针将^125^I粒子植入到气道周围的淋巴结或肿块内，根据治疗计划系统（treatment planning system, TPS）决定植入粒子数量，一般为5粒-15粒。粘膜下药物注射（今又生等）等根据操作常规进行。

### 1.4 统计学方法

采用统计软件SPSS 16.0进行数据分析。计量资料采用Mean±SD表示，采用*t*检验。以*P* < 0.05为差异有统计学意义。

## 2 结果

### 2.1 肿瘤分布部位

胸部CT所见16例肿瘤起源于主气管下端（3区）1例，右肺8例（包括右主支气管5区1例，右上叶支气管7例），左肺7例（包括左下叶支气管5例，左主支气管末端8区2例）。肿瘤呈周围型11例（68.8%），主要位于右上叶（6例）和左下叶肺内（5例）。5例（31.2%）位于中央型气道内。1例患者可有多个部位侵犯。位于肺内者以混合型多见（9/11, 82%），原发性较多（9/11, 82%）；而位于中央型气道者以管内型多见（9/16, 56%），转移性较多（11/16, 69%），次为外压型（5/16, 31%）。1个肿瘤可有多个部位侵犯（表 1）。

**1:** 胸部CT所见16例肿瘤分布部位 Distribution of 16 cases with tumor in chest CT scan

Distribution of tumor	Tumor type				Tumor origin		Total
	Intra-luminal	Mixted	Extrinsic compression		Primary	Metastasis	
2	0	0	1		0	1	1
3	2	0	2		1	3	4
5	2	2	1		2	3	5
7	0	0	1		0	1	1
8	5	0	0		2	3	5
RUL	1	5	0		5	1	6
LL lower	0	4	1		4	1	5
Total	10	11	6		14	13	27
The number in the left column represents the tumor region located in the airway. One patient may have several tumors existed in different regions of airway.RUL: right upper lobe; LLL: left lower lobe; CT: computed tomography.							

### 2.2 支气管镜介入治疗前后患者的状况

支气管镜介入治疗前胸部CT显示全肺不张4例（占25%），左右侧各2例，另有肺叶不张7例（43.8%），其中右上叶不张6例，左下叶不张1例。治疗后4例全肺不张均缓解（100.0%），3例肺叶不张完全缓解（42.9%），部分缓解和无效各2例（28.6%）。治疗后患者气道阻塞程度、KPS和气促评分均有明显改善（表 2）。

**2:** 支气管镜介入治疗前后患者的状况 Physical situations of patients after interventional bronchoscopy

Location	Cases	Before	After
Trachea	11	72±8	21±7^**^
Bronchus	5	51±10	16±6^**^
Segment bronchus	7	100±0	37±16^**^
KPS	16	67±5	80±4^*^
Shortness of breath score	16	2.5±0.3	1.3±0.2^**^
Comparison between before and after treatment, ^*^P < 0.05, ^**^P < 0.01. KPS: Karnofsky performance status.			

管内型肿瘤可全部清除，肺可完全或部分复张（图 1，图 2）。

**1:**
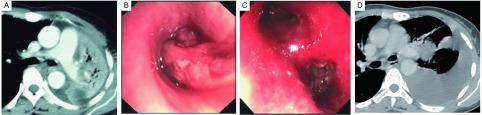
左下肺肉瘤样癌致左全肺不张。A：胸部CT显示左全肺不张，未见明确肿块；B：支气管镜显示左主支气管下端肿瘤将管腔完全堵塞；C：支气管镜下将肿瘤取出后，可见肿瘤来源于左下叶外后基底段，其他管口均畅通；D：胸部CT显示经支气管镜治疗后，左上肺复张，左肺门肿块伴胸腔积液。 Left lower atelectasis in patient with sarcoma. A: Left whole atelectasis without tumor showed in chest CT scan; B: Bronchoscopy found that left main bronchus was comletely obstructed by a tumor; C: The tumor origined from left outer basal segment when the tumor was removed by bronchoscope; D: Chest CT scan discovered that left lung reopened accompanied by left hilar tumor and pleural effusion after bronchoscopy.

**2:**
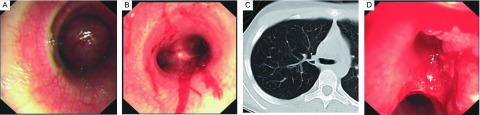
右上叶支气管纤维肉瘤致右全肺不张。A：气管下段管腔内可见类圆形肿瘤（管内型），管腔堵塞约90%，隆突及双侧支气管开口未见。B：在支气管镜下将气管下段肿瘤清除，管腔基本通畅；C：胸部CT发现右上叶支气管开口占位性病变；D：再次在支气管镜下将右上叶支气管开口肿瘤部分清除。 Right whole atelectasis in patient with right upper bronchus fibrosarcoma. A: Class round tumor (Introluminal) was found in lower part of trachea with 90% of obstruction. Carina and bilateral bronchial orifaces were not showed in bronchoscopy; B: Bronchoscopy found that tumor was removed in lower part of trachea with normal lumen; C: The tumor was occupied in right upper bronchus showed in chest CT scan; D: Partial tumor was removed through bronchoscopy in right upper bronchial oriface.

合并治疗：16例患者入院时均因病情较重，无紧急手术指征。11例仅行支气管镜介入治疗，其余则在此前后行化疗2例，支气管动脉介入治疗1例，经皮穿刺行氩氦刀治疗1例，放射性^125^I粒子植入1例。1例外压性病变腔内置入支架。1例儿童右上叶支气管肉瘤因支气管镜介入治疗术后复发较快，行右上叶手术切除。

### 2.3 随访

支气管镜介入治疗术后电话或门诊随访3个月-63个月，中位生存时间7个月，最长1例超过5年。

## 3 讨论

肺癌中的肉瘤最早于1864年Virchow提出，1992年更名为肉瘤样癌，2004年世界卫生组织肺肿瘤组织学分类将其定义为一类伴有肉瘤成分或肉瘤样分化的分化差的非小细胞肺癌^[8]^，原发于肺部的肉瘤样癌占肺部恶性肿瘤的0.3%-4.7%^[9]^。它含有肺癌和肉瘤两种成分，病理上又可分为多形性癌、梭形细胞癌、巨细胞癌、癌肉瘤及肺母细胞癌等5个亚型^[10]^。本组16例肺肉瘤患者有10例为肉瘤样癌，临床最多见。其次为纤维肉瘤。

李天女等^[11]^报道22例肺肉瘤样癌中，周围型占86.3%，中央型占13.7%，均表现为肺内较大肿块或结节，密度大部分不均匀，易侵犯邻近胸膜或胸壁，伴肺门、纵隔淋巴结转移。

本组肺肉瘤约2/3为周围型，主要位于右上叶和左下叶肺内，以混合型和原发性肿瘤多见（各占82%）。约1/3为中央型，且以管内型（56%）和转移性（69%）较多。其中全肺不张占25%，部分肺不张占43.8%，主要是因肿瘤堵塞大气道所致。

文献报道^[1]^肺肉瘤大多以手术切除为主，而气管镜下介入治疗仅见少量个案报告^[2, 3]^。从本文所见，约2/3患者伴有阻塞性肺不张，且93.8%为晚期患者，不适合紧急手术。但为了获得病理诊断，大多需先行支气管镜检查。既往支气管镜主要用于大气道内疾病的诊断，现在由于支气管镜介入治疗技术的发展, 更重要的是用于气道内疾病的治疗。

根据肿瘤在管腔内的位置，可采取不同的支气管镜介入治疗措施。管内型肿瘤可通过套取、冻取及APC等完全取出，阻塞的肺不张可完全缓解。而对腔内混合型肿瘤则需结合冻取及APC等将部分肿瘤取出，必要时结合内支架置入。治疗后患者气道阻塞程度、KPS和气促评分均有明显改善。管内型引起的4例全肺不张和3例肺叶不张治疗后均完全缓解，部分缓解和无效各2例（主要因肿瘤位于肺内，气管镜不能完全窥见）。

由于肺肉瘤对放/化疗均不敏感, 不宜作为首选治疗。如果肺内肿瘤较大、血运丰富，可先行支气管动脉栓塞，必要时结合氩氦刀消融治疗和间质放疗^[11-13]^。本组16例患者中11例仅行支气管镜介入治疗，无严重并发症发生。其余则在此前后行化疗2例，气管内支架置入、支气管动脉介入治疗、经皮穿刺行氩氦刀治疗和^125^I放射性粒子植入各1例。1例儿童右上叶支气管肉瘤因支气管镜介入治疗术后复发较快，行右上叶手术切除，术后随访4年未见复发。

本组16例患者中位生存时间7个月，最长1例超过5年。由此可见，支气管镜联合其它介入手段治疗肺肉瘤安全、有效，值得临床推广应用。
